# Conserved binding site in the N-lobe of prokaryotic MATE transporters suggests a role for Na^+^ in ion-coupled drug efflux

**DOI:** 10.1016/j.jbc.2021.100262

**Published:** 2021-01-08

**Authors:** Steven Castellano, Derek P. Claxton, Emel Ficici, Tsukasa Kusakizako, Robyn Stix, Wenchang Zhou, Osamu Nureki, Hassane S. Mchaourab, José D. Faraldo-Gómez

**Affiliations:** 1 Theoretical Molecular Biophysics Laboratory, National Heart, Lung and Blood Institute, National Institutes of Health, Bethesda, Maryland, USA; 2 Department of Molecular Physiology and Biophysics, Vanderbilt University, Nashville, Tennessee, USA; 3 Department of Biological Sciences, Graduate School of Science, The University of Tokyo, Bunkyo-ku, Tokyo, Japan

**Keywords:** secondary-active transporters, drug-efflux pumps, multidrug resistance, ion selectivity, molecular dynamics, double electron–electron resonance spectroscopy, DEER, double electron–electron resonance, MATE, multidrug and toxic-compound extrusion, MC, Monte Carlo, MD, molecular dynamics, MDR, multidrug resistance, TEV, tobacco etch virus

## Abstract

In both prokaryotes and eukaryotes, multidrug and toxic-compound extrusion (MATE) transporters catalyze the efflux of a broad range of cytotoxic compounds, including human-made antibiotics and anticancer drugs. MATEs are secondary-active antiporters, *i.e.*, their drug-efflux activity is coupled to, and powered by, the uptake of ions down a preexisting transmembrane electrochemical gradient. Key aspects of this mechanism, however, remain to be delineated, such as its ion specificity and stoichiometry. We previously revealed the existence of a Na^+^-binding site in a MATE transporter from *Pyroccocus furiosus* (PfMATE) and hypothesized that this site might be broadly conserved among prokaryotic MATEs. Here, we evaluate this hypothesis by analyzing VcmN and ClbM, which along with PfMATE are the only three prokaryotic MATEs whose molecular structures have been determined at atomic resolution, *i.e.* better than 3 Å. Reinterpretation of existing crystallographic data and molecular dynamics simulations indeed reveal an occupied Na^+^-binding site in the N-terminal lobe of both structures, analogous to that identified in PfMATE. We likewise find this site to be strongly selective against K^+^, suggesting it is mechanistically significant. Consistent with these computational results, DEER spectroscopy measurements for multiple doubly-spin-labeled VcmN constructs demonstrate Na^+^-dependent changes in protein conformation. The existence of this binding site in three MATE orthologs implicates Na^+^ in the ion-coupled drug-efflux mechanisms of this class of transporters. These results also imply that observations of H^+^-dependent activity likely stem either from a site elsewhere in the structure, or from H^+^ displacing Na^+^ under certain laboratory conditions, as has been noted for other Na^+^-driven transport systems.

The development of multidrug resistance (MDR) in major human microbial pathogens is an increasingly alarming public-health threat. Examples include *Mycobacterium tuberculosis*, *Plasmodium falciparum* (malaria), *Streptococcus pneumoniae*, *Staphylococcus aureus*, and HIV (1, 2, 3). Among other cellular mechanisms, MDR is greatly fostered by innate mechanisms that enable bacteria to expel a broad range of cytotoxic compounds out of the cell (4, 5). So-called MDR efflux pumps are the class of membrane transporters that specifically provide this kind of defense. These transporters deplete the cytosolic concentration of human-made antimicrobial drugs as well as natural immunologic compounds, thus protecting their intracellular targets. Accordingly, these membrane transporters are considered a potential target for pharmacological interventions against MDR (6).

Among the five known classes of bacterial MDR efflux pumps (5, 7, 8), the MATE family is the most recently recognized, and also the least characterized (9, 10, 11). This very limited understanding no doubt hampers the development of novel potential inhibitors against an important class of MDR proteins. From bacteria to plants to mammals, MATE transporters catalyze the efflux of a variety of xenobiotics, most of which are hydrophobic and weakly cationic. These compounds are able to enter the cell by diffusing across the cytoplasmic membrane, following their concentration gradient and often driven by the transmembrane electric field. MATEs counter this deleterious unregulated uptake by catalyzing the translocation of these substances back to the cell exterior (or the periplasm in gram-negative bacteria). To activate this uphill process, MATEs are believed to couple their molecular mechanism to the uptake of Na^+^ or H^+^, thus harnessing the electrochemical potential of these ions to power drug efflux.

Structurally, MATE transporters consist of two distinct domains of six transmembrane helices each (TM1–TM6 and TM7–TM12); these two domains, referred hereafter as N- and C-lobes, have the same topology with respect to the membrane plane but are symmetrically arranged with respect to its perpendicular (11, 12). Like other secondary-active transporters, MATEs appear to operate according to the alternating-access model (13). In this model, a transporter cycles between two major conformational states, each of which exposes ion and substrate binding sites to one or other side of the membrane, but not both concurrently. Multiple structures of seemingly outward-open MATEs have been resolved at varying resolutions (14, 15, 16, 17, 18, 19, 20, 21, 22). In these structures the N- and C-lobes adopt a V-like conformation, *i.e.*, they are in contact only on the intracellular side and project away from each other on the extracellular side, exposing a large cavity to the exterior. A recent structure of PfMATE (23) has revealed the first inward-open conformation of a MATE transporter, featuring a very similar V-like conformation but in the opposite orientation, with the cavity in between the N- and C-lobes accessible from the intracellular side.

As mentioned, MATEs are driven either by Na^+^ or H^+^ gradients, like most secondary-active transport systems. However, in many instances their ion specificity and stoichiometry remain to be conclusively established, and little is known about the mechanisms that couple ion binding to the interconversion between outward- and inward-open states or to substrate binding and release. NorM-VP and NorM-NG were the first MATEs reported to be driven by Na^+^ (24, 25), while more recently DinF-BH, NorM-PS, and VcmN have been described as H^+^-dependent (18, 22, 26). NorM-VC, by contrast, has been reported to be coupled to both Na^+^ and H^+^ (27); this dual specificity might also explain seemingly conflicting reports for ClbM (19, 28) and PfMATE (17, 23, 29). Confusingly, monovalent cations such as K^+^, Rb^+^, and Li^+^ have also been reported to influence substrate efflux in some cases (28, 30, 31, 32). It is important to note, however, that many of the functional studies of MATE transporters are based on live cells, rather than controlled reconstituted systems, implying that ion-dependent effects resulting from other membrane systems or from adaptive cellular mechanisms cannot be ruled out. Based on structures alone, it has also been difficult to clarify the matter of ion specificity, as the resolution of much of the data available does not permit an unambiguous interpretation. Likewise, crystallographic assays whereby electron-rich cations such as Cs^+^ and Rb^+^ are used as reporters can be misleading, as these ions do not in fact mimic Na^+^ but might displace H^+^ from electronegative sites.

In two recent studies of PfMATE and NorM-VC, we used molecular dynamics (MD) simulations (33) and double electron–electron resonance (DEER) spectroscopy (34) to identify a Na^+^ binding site in the N-lobe of these transporters; we also showed that it reflects a highly conserved sequence motif among prokaryotic MATEs (33). This Na^+^ site, subsequently corroborated by the high-resolution structure of inward-facing PfMATE (23), very likely explains the observation of Na^+^-dependent drug efflux among some transporters of this family, as well as others in the larger superfamily of MOP (multidrug/oligosaccharidyl-lipid/polysaccharide) exporters, such as the lipid-flippase MurJ (35). Interestingly, this Na^+^ site is also highly similar to those observed in other ion-driven transport systems with dual specificity for Na^+^ and H^+^ (36, 37, 38, 39, 40). The Na^+^ binding sites in those systems are in fact mildly H^+^ selective, but the large excess of Na^+^ over H^+^ in physiological conditions results in Na^+^ coupling (36, 41, 42). However, this “circumstantial” specificity implies that subtle variations in sequence or certain laboratory conditions might favor H^+^ binding instead. This promiscuity of the N-lobe binding site might thus explain some of the reports of H^+^-dependent activity among MATEs. Nonetheless, additional binding sites for H^+^ in the C-lobe or elsewhere in the structure have also been proposed as potential explanations for H^+^-coupling in this family (27, 29, 33, 34).

Here, we seek to validate or refute the proposed Na^+^ binding site (33, 34) by examining two additional MATEs, namely VcmN and ClbM, again using MD simulations and DEER spectroscopy. Along with PfMATE, VcmN and ClbM are the only prokaryotic MATEs whose molecular structures have been determined at atomic resolution, *i.e.* better than 3 Å (19, 22), permitting a clear-cut examination of the question of Na^+^ binding without ambiguities in regard to the protein conformation.

## Results and discussion

### Proposed doubly protonated states do not explain measured electron densities

**Figure 1:**
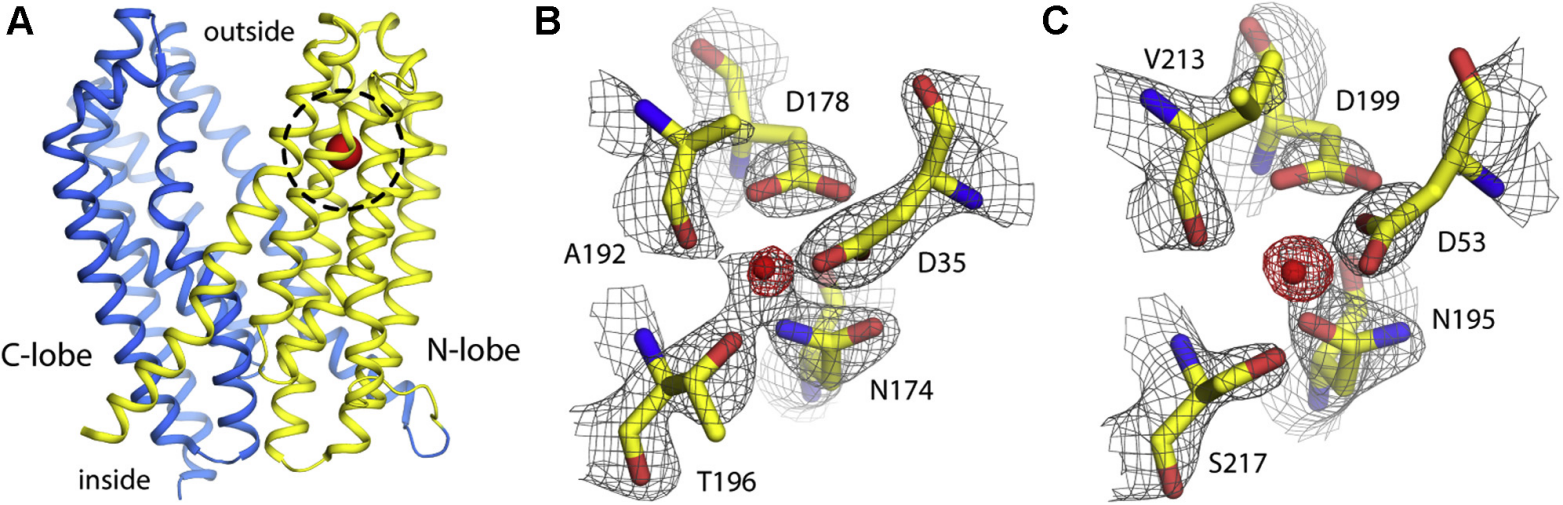
**Existing crystallographic data for VcmN and ClbM**. *A*, structure of VcmN determined by X-ray crystallography (PDB 6IDR) (22). N- and C-lobes (*yellow* and *blue*, respectively) adopt an outward-facing conformation. *B*, close-up of the putative ion-binding site in the N-lobe of VcmN, highlighting the residues proposed to define this site (sticks) along with the experimental 2F_o_–F_c_ density map, shown as a gray mesh at 2σ. Both Asp35 and Asp178 were proposed to be protonated in this configuration; a bound water molecule (*red sphere*) was proposed to explain the electron-density signal at the center of the site (22). The F_o_–F_c_ map that omits this putative water molecule is shown as a *red mesh*, at 7σ. *C*, Same as (*B*), based on available crystallographic data for ClbM (PDB 4Z3N) (28). The original interpretation of this data was also that protonated Asp53 and Asp199 coordinate a central water molecule (28). Note the sequence identity between VcmN and ClbM is only 23%. The NCBI Protein Conserved Domain Database lists over 25 sequence clusters for the MATE family; for example, VcmN and ClbM are in clusters cd13149 and cd13146, respectively. The mean, minimum, and maximum pairwise sequence identities within these clusters range from 35 to 50%, 25 to 40%, and 50 to 80%, respectively.

**Figure 2:**
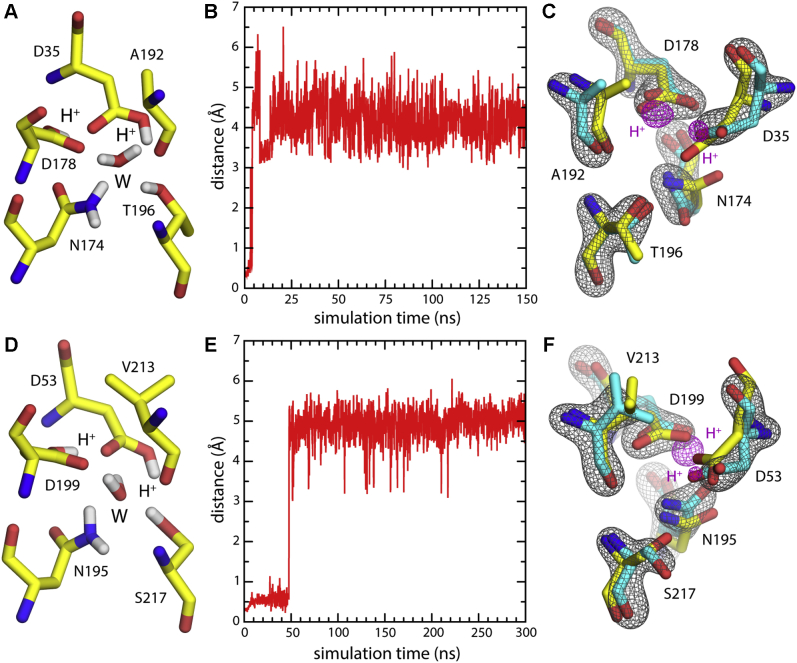
**MD simulations of VcmN and ClbM in the doubly protonated state originally assigned**. *A*, close-up of the putative H^+^ site in the N-lobe of VcmN, at the start of the simulation. As proposed (22), both D35 and D178 are protonated and a water molecule (W) occupies the center of the site. Nonpolar hydrogens are omitted for clarity. *B*, time series of the distance between the center of the site and the nearest water molecule in the simulation system; the time window corresponds to an early equilibration stage, when conformational restraints are still applied to the protein to preserve the experimental geometry; water dissociates soon after it is allowed to move freely. *C*, overlay of the crystal structure (PDB 6IDR, *yellow*) with a 3D density map (*mesh*) derived from a subsequent 300-ns trajectory, unrestrained (*gray*: protein; purple: H^+^ at D35 and D178), as well as with a randomly selected simulation snapshot (*cyan*). *D–F*, Same as (*A–C*) for the H^+^-bound state proposed for ClbM (PDB 4Z3N) (28).

Based on their crystallographic data for outward-facing VcmN (PDB 6IDR), Kusakizako *et al.* (22) concluded that a functional H^+^-binding site exists in the N-lobe of the transporter, approximately halfway between the membrane midplane and the extracellular surface of the protein (Fig. 1*A*). The proposed binding site is formed by four side chains, namely Asp35 on TM1, Asn174 and Asp178 on TM5, and Thr196 on TM6; the backbone carbonyl of Ala192, also in TM6, is the fifth component (Fig. 1*B*). A strong spherical electron-density signal not attributable to the protein was also detected at the center of this site (Fig. 1*B*). Kusakizako *et al.* interpreted this signal as a bound water molecule, which they suggested mediates a network of hydrogen-bonding interactions whereby both Asp35 and Asp178 are protonated (Fig. 2*A*). This interpretation is identical to that previously made for outward-facing PfMATE by Tanaka *et al.* (17).

Similarly, for outward-facing ClbM (PDB ID 4Z3N), Mousa *et al.* (28) reported a proton-binding site comprising Asp53, Asn195, Asp 199, Ser217, and Val213, in positions equivalent to those above (Fig. 1*C*). The serine and valine at positions 217 and 213 (196 and 192 in VcmN) are common substitutions for Thr and Ala in the MATE family (33). As for PfMATE and VcmN, a spherical electron-density signal is clear in the center of this site (Fig. 1*C*). Mousa *et al*. assigned this density to a bound water molecule, presumably following Tanaka *et al*., and accordingly interpreted this structure of ClbM as doubly protonated, at Asp53 and Asp199 (Fig. 2*D*)

In our previous study of outward-facing PfMATE (33), we reported MD simulations showing that the doubly protonated configuration proposed by Tanaka *et al.* (17) cannot sustain a bound water molecule and therefore does not explain the experimental electron-density map. Here, we report analogous MD trajectories for VcmN and ClbM, with which we examine the interpretation put forward by Kusakizako *et al.* (22) and Mousa *et al.* (28) (Fig. 2). In both cases we find that the putative water molecule rapidly exits the binding site in the early stages of the simulation, even though at that point the internal geometry of the site is still loosely restrained as observed in the crystal structure (Fig. 2, *B* and *E*). Thereafter, neither site becomes reoccupied by any other water molecule in the simulation system (containing >10,000 molecules). Ultimately the side-chain geometry in both binding sites shows significant changes relative to the X-ray structures, but the deviations are not drastic (Fig. 2, *C* and *F*). That is, as we noted for PfMATE (33), while the proposed doubly protonated states appear approximately compatible with the experimental data, this interpretation fails to explain the most prominent feature of the electron-density maps for both VcmN and ClbM, namely the strong signal at the center of the site.

### The N-lobe in VcmN and ClbM features an occupied Na^+^ binding site

In view of the discrepancy between our simulation results and the original interpretation of the structural data for VcmN and ClbM, we set out to examine the notion that the unexplained electron-density signal corresponds to a Na^+^ ion. As mentioned, Na^+^ is known to be the coupling ion for several MATE antiporters (24, 25, 27, 34, 43). Indeed, the overarching conclusion from our previous simulation study of outward-facing PfMATE (33) is that the binding site in the N-lobe is a Na^+^ site—a conclusion later verified by the crystal structure of inward-facing PfMATE, and simulations based on that structure (23). In our previous study we also presented bioinformatic evidence that this site is broadly conserved among prokaryotic MATEs and highlighted ClbM (33). A recent simulation study of ClbM has followed that prediction too (44).

It is important to note that water and Na^+^ cannot be distinguished at the resolution of the experimental structural data obtained for VcmN (2.5 Å) and ClbM (2.7 Å), because both appear as spherical signals reflecting the same number of electrons. Therefore, Na^+^ is just as plausible based on visual inspection of this data, for example, in F_o_–F_c_ omit maps (Fig. 1, *B* and *C*). Nonetheless, it is worth noting that the density signal in question is surrounded by five polar contacts arranged in a trigonal biplanar geometry, which is akin to that of known Na^+^ sites in other membrane transport proteins (35, 42, 45, 46, 47, 48). This interpretation would also be consistent with the calculated "valence" of these sites (49). This valence is a statistical metric of the similarity between a given ion binding site and others in known protein structures, in terms of their geometry and the number of coordinating ligands (Methods); the ion whose charge best approximates the calculated valence is statistically the most probable. In this case, the valence of these sites suggests an ideal match for Na^+^ (*i.e.*, a valence of 1.0) but not K^+^ or Ca^2+^ (valences of 3.7 and 1.6, respectively).

**Figure 3:**
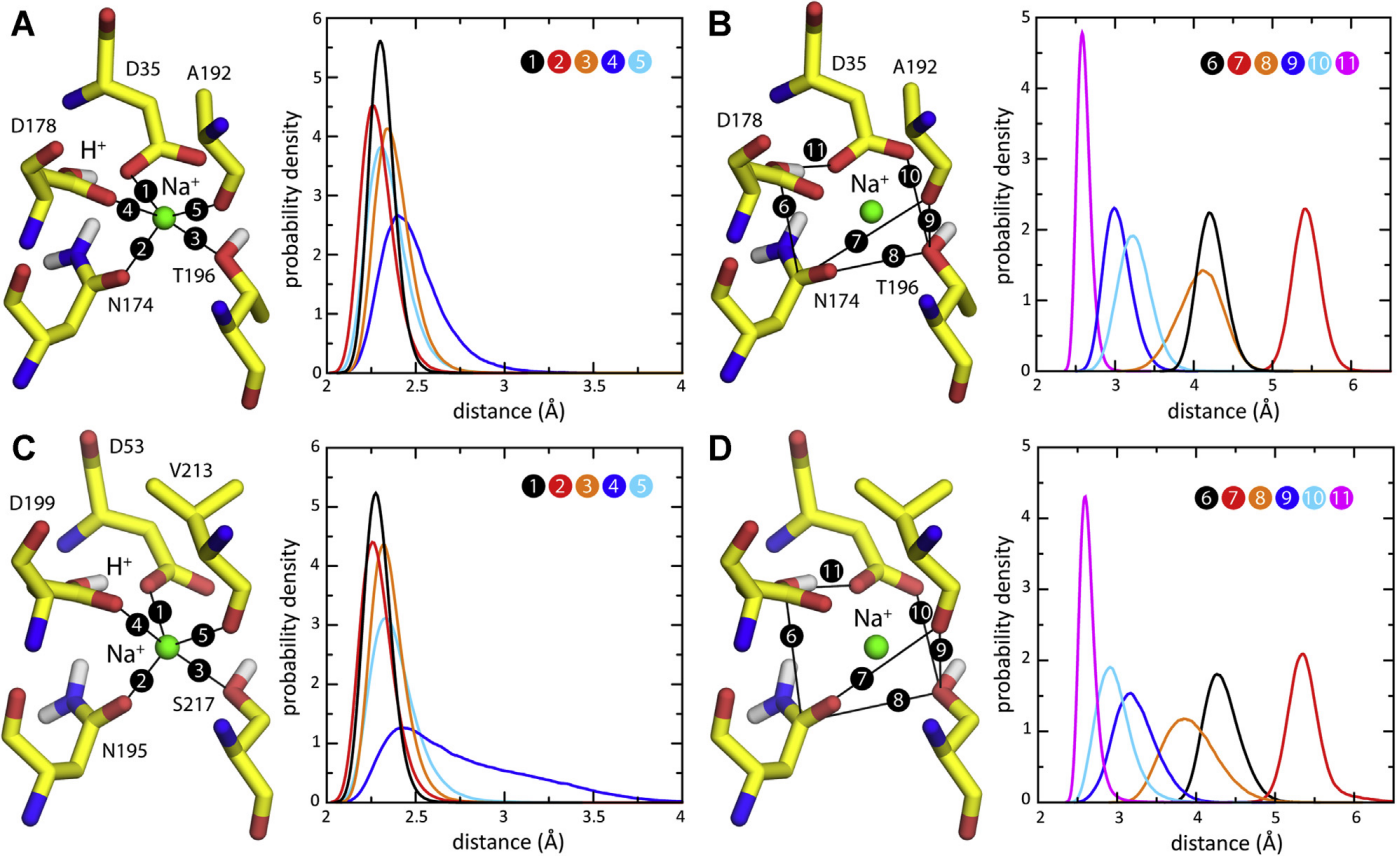
**Simulations of VcmN and ClbM with Na**^**+**^**bound to the N-lobe site**. *A*, close-up of the binding site in VcmN, occupied by Na^+^ (*green spheres*) at the beginning of the simulation. Note only D178 is protonated, forming a carboxyl-carboxylate pair with D35. The Na^+^ ion is coordinated by five ligands in the protein (numbered from 1 to 5). The adjacent plot shows calculated probability distributions for each of these distances, derived from an unrestrained simulated trajectory of 1 μs. *B*, same as (*A*), for six protein–protein distances defining the geometry of the binding site (numbered from 6 to 11). *C*, *D*, same as (*A*, *B*), for the site in the N-lobe of ClbM, bound to Na^+^, from an unrestrained simulated trajectory of 1 μs.

**Figure 4:**
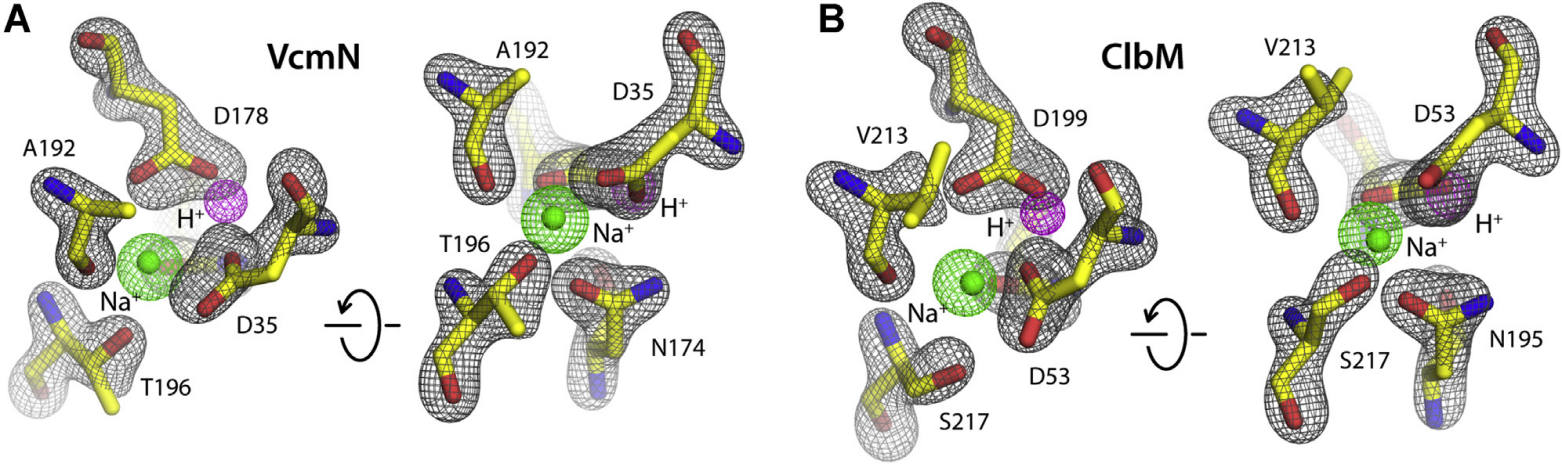
**Experimental and simulation binding-site geometries for the proposed Na**^**+**^**-bound state**. *A*, two close-up views of the Na^+^ binding site in the N-lobe of VcmN. The arrangement of the protein atoms and the object in the center of the site is that reported in PDB 6IDR (22). Overlaid is a calculated 3D density map (*mesh*) for the same elements, from our molecular dynamics simulation of the Na^+^ bound state (*gray*: protein; *green*: Na^+^; *purple*: H^+^ at D178). *B*, same as (*A*), for the Na^+^-binding site in ClbM (PDB 4Z3N) (28).

To evaluate whether the experimental data for outward-facing VcmN and ClbM indeed reflect an occupied Na^+^ binding site within the N-lobe, we followed the same methodology employed for PfMATE (Methods) (33). First, we utilized continuum-electrostatic calculations to identify the most probable protonation state for each of the ionizable sidechains in the protein, in the presence of the putative Na^+^ ion. We then prepared a simulation system for this most favored configuration and calculated a 1-μs MD trajectory for each transporter. The continuum-electrostatic calculations showed that with Na^+^ occupying the site, Asp178 and Asp199 in VcmN and ClbM, respectively, are highly likely (>99.9%) to be protonated in the physiological pH range, while Asp35 and Asp53 have very low (<0.1%) protonation probabilities. That is, in this proposed configuration one H^+^ coexists with Na^+^ in the binding site. Interestingly, the acidic side chain at the position of Asp178 in VcmN and Asp199 in ClbM is frequently replaced by Asn across the MATE family (33); thus, it is very possible that H^+^ plays a structural or regulatory role in some MATEs. In any case, the MD simulations carried out for this configuration lend clear support to the notion that the unexplained density signal in the N-lobe site is indeed a Na^+^ ion in both VcmN and ClbM (Figs. 3 and 4). Probability distributions derived from the 1-μs trajectories for a set of defining protein–protein and ion–protein interaction distances showed no evidence of Na^+^ dissociation and every indication of a stable configurational ensemble (Fig. 3); moreover, that ensemble closely resembles the experimental protein geometry for both VcmN and ClbM (Fig. 4).

### N-lobe Na^+^-binding site is selective against K^+^

The simulation data discussed above clearly support the hypothesis that the N-lobe of VcmN and ClbM feature a Na^+^-binding site, which appears to be broadly conserved across prokaryotic MATEs (33). An expected feature of a functionally significant ion-binding site in a membrane transporter is its selectivity. In this case, the proposed Na^+^ site ought to be selective against other physiologically relevant cations, specifically K^+^, which is similarly abundant. This selectivity can be evaluated computationally, by quantifying the free-energy cost (or gain) associated with replacing Na^+^ by K^+^ when bound to the protein, relative to the analogous value in the bulk solution. That is, we quantified the difference in the binding free energy of these two cations. For completeness, we considered two cases: one in which the geometry of the binding site is restricted to that captured in the crystal structure, *i.e.*, the ion coordination mode is constant, and another in which the geometry can adapt freely to the larger K^+^ ion, *i.e.*, the coordination state can change.

**Figure 5:**
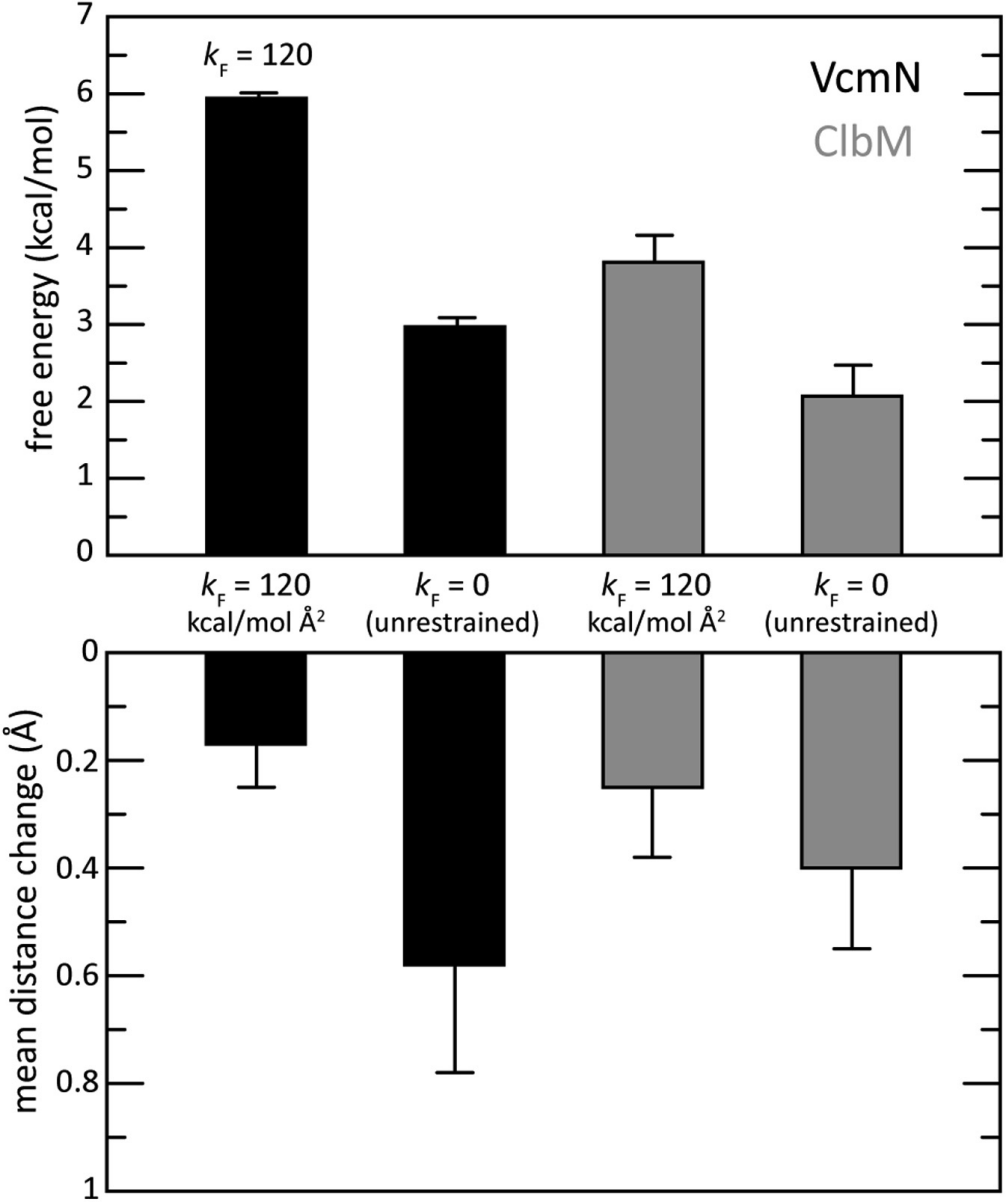
**Selectivity of the Na**^**+**^**-binding site in the N-lobe of VcmN and ClbM against K**^**+**^. Top, calculated free-energy difference between a hypothetical K^+^-bound state and the Na^+^-bound state proposed here, based on FEP simulations, for both VcmN (*black*) and ClbM (*gray*). Two cases are considered for both proteins: one in which protein–protein distance restraints restrict the degree to which the geometry of the binding sites can adapt to K^+^, relative to those shown in Figure 3 (the restraint force constant *k*_F_ is indicated); and another in which the geometry of the binding sites can adjust freely. The lower panel quantifies the mean change in the four distances considered in each case (numbered 6, 8, 10 and 11 in Fig. 3).

Consistent with the notion that the structures of both VcmN and ClbM capture Na^+^-bound states, we found that the selectivity of the site against K^+^ was maximal when the experimental geometry was strongly enforced, and that this selectivity was lower when the protein is allowed complete flexibility (Fig. 5). Even then, however, there remains a significant free-energy penalty for K^+^ displacement of Na^+^, namely ∼2–3 kcal/mol. This value translates into a preference for Na^+^ of 50- to 100-fold, implying that Na^+^ would compete out K^+^ under any physiological condition. Insofar as selectivity indicates functionality, this analysis thereby suggests that the proposed N-lobe site is a functional Na^+^ site.

### DEER spectroscopy confirms Na^+^-dependent conformational changes in VcmN

To experimentally evaluate the computational results described above we utilized DEER spectroscopy. DEER signals arise from the distance-dependent dipolar interaction between two spin labels incorporated at specific sites in a protein *via* engineered cysteines (50). These signals can be transformed into probability distributions for the distance between spin labels, *P*(*r*), with an associated confidence band (51, 52). Through systematic analyses of signals from multiple spin-label pairs, studies of membrane transporters (53, 54, 55, 56, 57, 58, 59), including several MATEs (29, 34), have examined how their conformational equilibria are modulated by ions and substrates. This modulation is manifested by shifts in the populations of the discrete distance components of *P*(*r*), reflecting changes in the conformational energy landscape of the protein (60, 61, 62). Here, we seek to examine a basic prediction: if the N-lobe harbors a Na^+^-binding site, addition of Na^+^ to VcmN should alter its conformational dynamics and therefore reveal changes in the *P*(*r*) distributions derived from DEER data.

**Figure 6:**
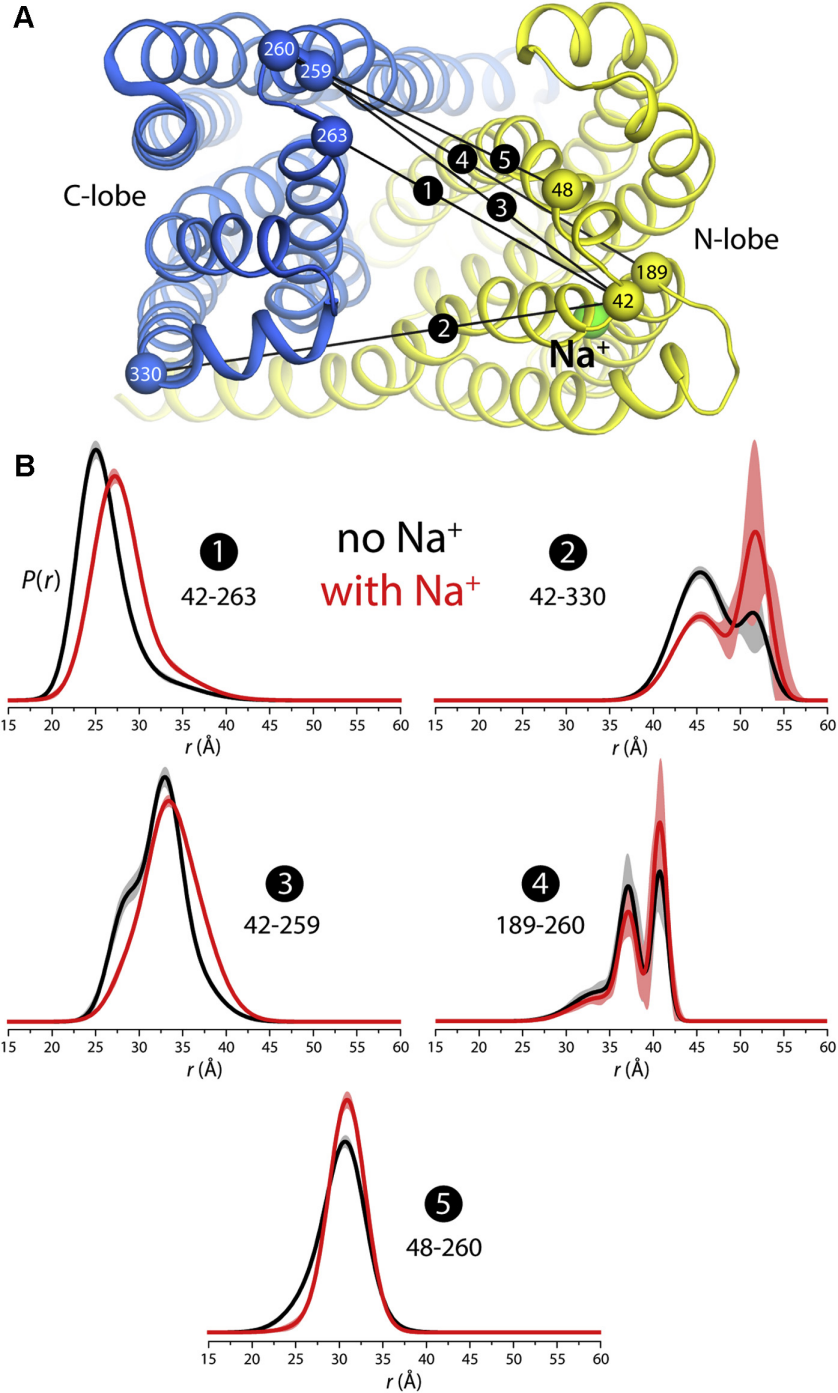
**Conformational dynamics of VcmN is modulated by Na**^**+**^. *A*, crystal structure of outward-facing VcmN (PDB 6IDR), viewed from the extracellular side. *Blue/yellow spheres* and connecting *black lines* mark the Cα atoms of the residue pairs for which spin-labels pairs were engineered. The Na^+^-binding site in the N-lobe is also highlighted (*green*). *B*, probability distributions, *P*(*r*), for each of the distances between spin labels depicted in panel (*A*), derived from measured EPR spin-echo time signals in each cased. Results are shown for experiments carried out in the absence and in the presence of 80 mM NaCl (*black* and *red*, respectively), at pH 7.5. Confidence bands for each *P*(*r*) are shown alongside, at 2σ (95%) (*gray* and *light red*, respectively).

On a mutant background of VcmN devoid of endogenous cysteines (22), five MTSSL spin-label pairs were introduced on the periplasmic side of protein as reporters of the distance between the N- and C-lobes (Fig. 6*A*). The specific choice of sites for labeling was informed in part by our previous study of NorM-VC, a prokaryotic MATE homologous to VcmN that demonstrated sensitivity to Na^+^ binding to the proposed site in the N-lobe (34). Importantly, for every pair of spin labels, that introduced in the N-lobe is in proximity to the proposed Na^+^ site.

Distance distributions were obtained in the absence and presence of 80 mM Na^+^. In both conditions, the *P*(*r*) curves reflect broad, oftentimes multicomponent distance distributions consistent with a substantial degree of heterogeneity, seemingly resulting from the dynamics of the spin labels, the protein backbone, or both (Figs. 6*B* and S1). Nonetheless, and importantly, addition of Na^+^ induced a discernable shift toward longer distances for all pairs, due to a reweighting of short and long-distance components. These shifts are well above the uncertainty of the *P*(*r*) curves and are more clearly noticeable when one spin label is very close to the proposed binding site (*e.g.*, 42–330 *versus* 48–260). Interestingly, a similar pattern was observed in the abovementioned studies of NorM-VC upon Na^+^ recognition (34). While more detailed studies such as those carried out for NorM-VC and PfMATE (29, 34) will be required to establish the relationship of Na^+^ binding to transport, the evidence presented here strongly suggests that a Na^+^-binding site that modulates the transporter dynamics does exist in the N-lobe of VcmN.

## Conclusions

Like most other antiporters, MATE transporters must be able to catalyze two distinct transport reactions. In both reactions, the protein must carry a cargo from one side of the membrane to the other, through the interconversion between inward and outward-facing conformations—the so-called alternating-access model (17, 23). That this interconversion occurs only upon recognition of either the substrate or the coupling ions, but not with both simultaneously, nor in the apo state, is essential; it is this exclusivity that permits the antiporter to sustain its uphill drug-efflux activity with the free-energy gain derived from downhill ion uptake. While many open questions remain about this complex mechanism, it is clear that to identify the location of the binding sites for ions and substrates is a key step toward a better understanding. In our previous study, computer simulations of an archaeal MATE and a family-wide bioinformatic analysis led to the hypothesis that many prokaryotic MATEs feature a Na^+^-binding site in the N-terminal domain, which seems likely to be involved in the mechanism that couples substrate efflux to ion uptake; interestingly, this site is occasionally found in proteins of the larger MOP superfamily, *e.g.*, the lipid-flippase MurJ. Here, we have corroborated that hypothesis for two additional MATEs, using computational and experimental methods. Our results thus further underscore the potential mechanistic significance of this conserved motif. Our calculations also indicate that this site strongly favors Na^+^ against K^+^, as one might expect for a functional site in a secondary-active transporter, but it is worth noting that its amino-acid makeup and geometry also appear compatible with H^+^ binding, possibly explaining reports of dual specificity in this class of systems. Going forward, it will be important to more precisely quantify these preferences through competitive binding assays in controlled experimental conditions (39, 42, 63, 64), so as to conclusively establish the ion specificity and stoichiometry of this class of systems and facilitate the design and interpretation of new functional assays.

## Methods

### Crystallographic data analysis

Calculated F_o_–F_c_ omits map and atomic models are based on published structure factors as deposited in the Protein Data Bank, ID codes 6IDR (VcmN) (22), and 4Z3N (ClbM) (28). The crystallographic data were processed with PHENIX version 1.8.4 (65, 66) and Coot (67). The valence of the N-lobe site was calculated as ∑_i_(*R*_i_/*R*_o_)^-*N*^ (49), where *i* denotes each of the ligand–protein contacts within 3 Å, *R*_*i*_ is the contact distance in each case, and *R*_o_ and *N* are empirical parameters with the following values: 1.6 and 4.29 for Na^+^, 2.276 and 9.1 for K^+^, and 1.909 and 5.4 for Ca^2+^.

### Protonation states of ionizable residues

A Monte Carlo (MC) algorithm was used to identify the most likely set of protonation states reflected by the experimental structures, based on a continuum-electrostatics framework (68). Electrostatic-energy evaluations used the Poisson equation solver PBEQ in CHARMM 39b2 (69). The membrane was represented with a rectangular slab with dielectric constant of 2, while that of the protein interior was set to 4. The surrounding solution and solvent-accessible cavities within the protein were assigned a dielectric constant of 80. Atomic charges were those in the CHARMM36 force field (70) and atomic radii were those of Nina *et al.* (71). Only Asp, Glu, and His side chains were considered in this analysis (Lys and Arg were assumed to be protonated). The MC algorithm sampled a diverse set of all possible combinations of protonation states for these residues, favoring those with the best electrostatic energy at a given pH. From this “trajectory,” the protonation probability *P* for a given side chain was estimated from the frequency with which the protonated state was observed. Two cases were studied, either with or without a Na^+^ ion occupying the binding site in the N-lobe. (The bound Na^+^ ion was represented with a charge of +1e and a radius of 1.66 Å.) In both cases, most side chains favored their standard protonation state for the pH values considered, generally in the range of 5.0–6.0 and 7.0–7.5 (roughly representing the pH of the periplasm and cytoplasm, respectively). For example, the calculated *P* for His352 in VcmN is 47% at pH 5 and 4% at pH 7, consistent with its location in an exposed loop on the extracellular side. Likewise, the calculated *P* for Glu124 in VcmN, which is also exposed, is 10% at pH 5 but 0.2% at pH 7.

### Molecular dynamics simulations

All MD simulations were carried out with NAMD version 2.7 (72), using the CHARMM36 force field for proteins and lipids, with an NBFIX correction for sodium–carboxylate interactions (46, 70, 73). Temperature and pressure were kept constant at 298 K and 1 atm, respectively. A time step of 2 fs was used. Electrostatic interactions were calculated using the Particle-Mesh-Ewald method with a real-space cutoff of 12 Å. The same cutoff distance was used for van der Waals interactions, with a switching function turned on at 10 Å. Periodic boundary conditions were used. All simulations were based on PDB ID codes 6IDR and 4Z3N for VcmN and ClbM, respectively. Crystallographic water molecules within the protein solvent-excluded surface were retained, and additional water molecules with the protein were added with DOWSER. The resulting structures were briefly energy-minimized with CHARMM (500 steps) and embedded in a preequilibrated lipid bilayer (1-palmitoyl-2-oleoyl-sn-glycero-3-phosphocholine) using GRIFFIN (74). The surrounding solution contained 100 mM NaCl and counterions to neutralize the total charge of the systems. Altogether, the simulation systems each comprised ∼85,000 atoms in an orthorhombic box of approximately 90 Å by 90 Å by 120 Å. All simulations included an initial equilibration protocol in which the dynamics of the protein–ligand (water/Na^+^) complex is restricted, primarily through internal-conformation restraints, to a degree that is gradually reduced in a series of stages; this relaxation protocol comprised 150 ns for VcmN and 300 ns for ClbM. Thereafter, the structural dynamics of the protein–ligand complex was entirely unrestricted. For the Na^+^-bound states, we calculated trajectories of 1 μs each. For the doubly protonated states, we calculated trajectories of 300 ns.

### Free-energy of ion selectivity

MD simulations were also used to evaluate the free-energy difference between the proposed Na^+^-bound state and a hypothetical K^+^-bound configuration. This free-energy value was evaluated for both VcmN and ClbM in two different cases: one in which the geometry of the binding site was biased to remain as observed in the crystal structure, using a set of protein–protein distance restraints, each represented by a flat-harmonic potential of force constant 120 kcal mol^-1^ Å^-1^; and another in which the binding site was permitted to freely adapt to K^+^. The free-energy perturbation module of NAMD (72) was used to induce the alchemical transformation of the Na^+^ ion bound to the protein into K^+^ and, concurrently, of a K^+^ ion in solution into Na^+^, recording the resulting free-energy change. (The ion transformed in solution was kept away from the protein and membrane using a boundary potential, so that |z| > 35 Å.) The process was then reversed and the free energy recomputed. Each transformation was made gradually, using a parameter λ that scales up (or down) the van der Waals radius of the ion, in 50 consecutive simulations of 300 ps each (the initial 100 ps were discarded as equilibration time). Mean values from the forward and backward transformations are reported along with their difference. To describe the change in the geometry of the binding site upon transformation of Na^+^ into K^+^, we evaluated the time course of each of the abovementioned protein–protein distances, $d_{i}$, and quantified $\Delta D=\left(1/N\right)\overset{N}{\underset{i=1}{\sum }}\left|\Delta d_{i}\right|$ and $\Delta \Delta D={\left[(1/N\left(N-1\right))\overset{N}{\underset{i=1}{\sum }}{\left(\left|\Delta d_{i}\right|-\Delta D\right)}^{2}\right]}^{1/2}$, where $\Delta d_{i}=\langle d_{i}\;\left(Na^{+}\right)\rangle -\langle d_{i}\;\left(K^{+}\right)\rangle$ and *N* is the number of distances considered.

### Mutagenesis, purification, and spin labeling

All mutants of VcmN were created by site-directed mutagenesis. All endogenous cysteines (Cys154, Cys314, Cys366, and Cys397) were replaced with alanines, and then the double cysteines were introduced into the cysteineless wild-type background. The protein preparation was performed essentially as described previously with modifications (22). Mutant VcmN was cloned into a modified pET-28a vector encoding a C-terminal tobacco etch virus (TEV) protease cleavage site followed by a His_6_-tag. *Escherichia coli* C41(DE3) Rosetta strain harboring pRARE was transformed with VcmN plasmid and cultured in LB media containing 30 μg mL^−1^ kanamycin. Protein expression was induced with 0.4 mM isopropyl β-_D_-thiogalactopyranoside (IPTG) when the culture reached an absorbance at 600 nm (A_600_) of 0.5–0.8 and allowed to continue for 20 h at 20 °C. Cells were harvested by centrifugation at 5000*g* for 10 min and disrupted by a Microfluidizer (Microfluidics) at 15,000 psi for 2–3 passes. The lysate was centrifuged at 28,000*g* for 30 min, and the membrane fraction was isolated from the supernatant by ultracentrifugation at 125,000*g* for 1 h. The membrane fraction was solubilized in buffer (20 mM Tris-HCl, pH 8.0, 300 mM NaCl, 20 mM imidazole) with 1.5% n-dodecyl-β-D-maltoside (DDM) for 1 h at 4 °C and subjected to ultracentrifugation to remove debris. The supernatant containing solubilized VcmN was mixed with 5 ml of Ni-NTA resin (QIAGEN), equilibrated with buffer A (20 mM Tris-HCl, pH 8.0, 300 mM NaCl, 0.1% DDM) containing 20 mM imidazole, for 1 h. The resin was washed with ten column volumes of buffer A containing 30 mM imidazole, and then the protein was eluted with buffer A containing 300 mM imidazole. His-tagged TEV protease (prepared in-house) was added to the eluted fraction, and the mixture was dialyzed against buffer (20 mM Tris-HCl, pH 8.0, 300 mM NaCl, 0.02% DDM) overnight at 4 °C. The solution was reloaded onto the Ni-NTA resin, and the flow-through fraction was collected. The solution was concentrated by an Amicon Ultra centrifugal filter (50 kDa molecular weight cutoff, Millipore) and applied to a Superdex200 Increase 10/300 Gl (GE Healthcare) gel-filtration column equilibrated with 20 mM Tris-HCl, pH 8.0, 100 mM NaCl, 0.1% DDM buffer.

Purified VcmN double-Cys mutants obtained from SEC were concentrated to 0.5 ml and then incubated with 2 mM DTT on ice for 30 min. The reducing agent was removed by buffer exchange, using a 5 ml Sephadex G-25 desalting column (GE Healthcare) equilibrated with 50 mM Tris-MES, pH 7.5, 0.05% DDM, and 10% (v/v) glycerol. No Na^+^ was included in the buffer. The sample was labeled by two incubations with a 30-fold molar excess of 1-oxyl-2,2,5,5-tetramethylpyrroline-3-methyl methane thiosulfonate (MTSSL, Enzo Life Sciences), a thiol-specific spin label reagent, at 4 °C in the dark over a 4-h period. After the third addition of the spin label, the sample was incubated on ice for 12 h. The unreacted spin label was removed by gel filtration chromatography on a Superdex 200 Increase 10/300 GL (GE Healthcare) column, in the same buffer. The peak fractions were combined and concentrated with an Amicon Ultra filter (100 kDa molecular weight cutoff) for the EPR and DEER analysis.

### Double electron–electron resonance spectroscopy

EPR spectra were collected at 23 °C with a Bruker EMX spectrometer (X-band, 9.5 GHz), at an incident power of 10 mW and a 1.6 G modulation amplitude. Distance measurements were performed with a Bruker E580 pulsed EPR spectrometer at the Q-band frequency (34 GHz), by employing a standard four-pulse protocol at 83 K (50). Pulse lengths were 10–12 ns (π/2) for the probe pulse and 40 ns for the pump pulse. The frequency separation between probe and pump pulses was 63 MHz. DEER experiments were performed in the absence and presence of 80 mM NaCl. The samples for the DEER analysis were cryo-protected with 25% (w/v) glycerol and flash frozen in liquid nitrogen. The DEER signals obtained under different conditions for the same spin-labeled pair were analyzed globally with in-house written software operating in the Matlab (MathWorks) environment (51, 52). The fitting routine assumed that the distance distribution *P*(*r*) is a sum of Gaussians. The number of Gaussians required to sufficiently describe *P*(*r*) was statistically determined by Bayesian analysis. Confidence bands, which are reported at 2σ (95%), are shown about the best fit line for *P*(*r*) of each biochemical state. These bands depict the estimated uncertainty in *P*(*r*) and are derived from the error in the best fit parameters for each Gaussian component, which is associated with the noise and background correction in the fitting of the time domain spin echo decay.

## Data availabilty

All relevant data are contained within this article and in the supporting information.

## Conflict of interest

The authors declare that they have no conflicts of interest with the contents of this article.
