# Supplementary material for: Conserved binding site in the N-lobe of prokaryotic MATE transporters suggests a role for Na+ in ion-coupled drug efflux
Source: J Biol Chem. 2021 Jan 8;296:100262. doi: 10.1016/j.jbc.2021.100262 (PMC7949106; doi:10.1016/j.jbc.2021.100262)
Supplement: Supplementary file 1 — Figure S1 [file mmc1.pdf]

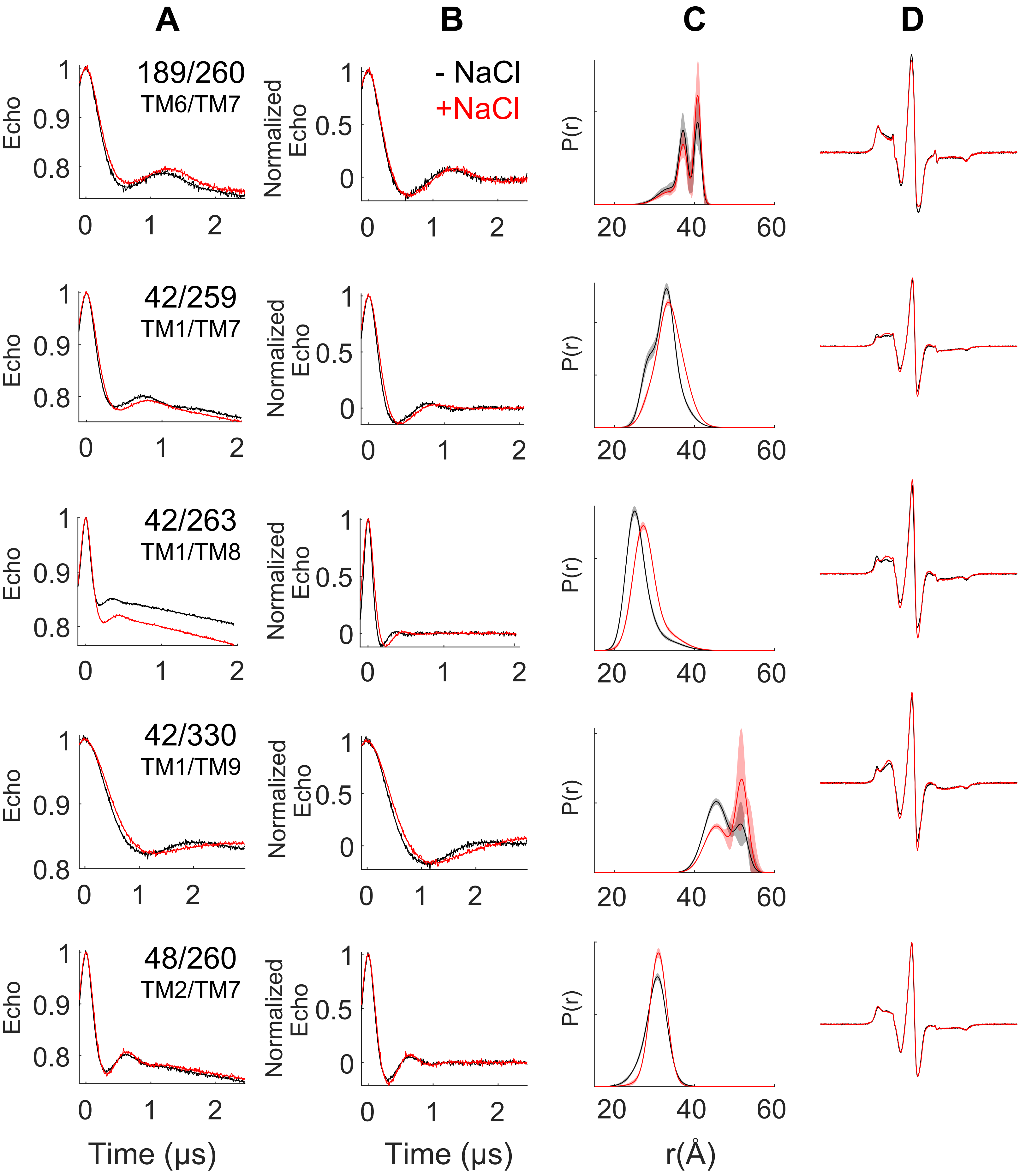

Figure S1. DEER analysis of VcmN double mutants. (A) Primary DEER signal and associated fits, (B) normalized and background-corrected signals with fits (C) the distance distribution P(r) with confidence bands (2σ) and (D) CW-EPR spectra.
